# Ordered phase transformation and Cu doping effects in room-temperature ferromagnetic Sr_3_YCo_4_O_10.5+*δ*_

**DOI:** 10.3389/fchem.2022.1073946

**Published:** 2022-12-08

**Authors:** Lingqi Ren, Xiaodong Zhang, Xiaoli Du, Jianlu Wang, Lan Yu

**Affiliations:** ^1^ Faculty of Materials Science and Engineering, Kunming University of Science and Technology, Kunming, China; ^2^ State Key Laboratory of Infrared Physics, Shanghai Institute of Technical Physics, Chinese Academy of Sciences, Shanghai, China

**Keywords:** ordered phase transformation, electrical transport, room-temperature ferromagnetism, ceramics, Cu content

## Abstract

Sr_3_YCo_4_O_10.5+*δ*_ (314-SYCO), with an unusual ordered structure and a high Curie temperature (*T_c_* ≈ 335 K), is attracting increasing attention. Herein, to improve the electrical performance of 314-SYCO, Cu-doped Sr_3_YCo_4−*x*_Cu_*x*_O_10.5+*δ*_ (*x* = 0–0.8) ceramics were prepared using a solid-state reaction method. Systematic research was conducted on both the ordered phase transformation and the effects of Cu doping on the microstructure, electrical transport characteristics, and magnetic properties. For *x* = 0–0.4, the (103) and (215) planes were observed and combined with Rietveld refinement results for the X-ray diffraction data, confirming the formation of ordered tetragonal Sr_3_YCo_4−*x*_Cu_*x*_O_10.5+*δ*_. This phase was formed with a mass gain of ∼0.8% and heat released at ∼1,042°*C*. With increasing Cu content, the concentration of hole carriers also increased, leading to a substantial reduction in electrical resistivity. The electrical resistivity decreased by 92–99% at 300 K. The polycrystalline materials have semiconducting behaviour with a three-dimensional Mott variable-range hopping mechanism. For the magnetic properties, a Hopkinson peak was observed at 319 K, and the *T_c_* was approximately 321 K for *x* = 0. The magnetisation and *T_c_* decreased with increasing Cu content, and a *G*-type antiferromagnetic-to-ferromagnetic phase transition occurred due to the spin state change for some Co^3+^ ions from high/intermediate spin to low/intermediate spin. These results lay the groundwork for refinement of the sintering procedure and doping parameters to enhance the performance of 314-SYCO in the context of current applications such as microwave absorbers and solid oxide fuel cell cathodes.

## 1 Introduction

**FIGURE 1:**
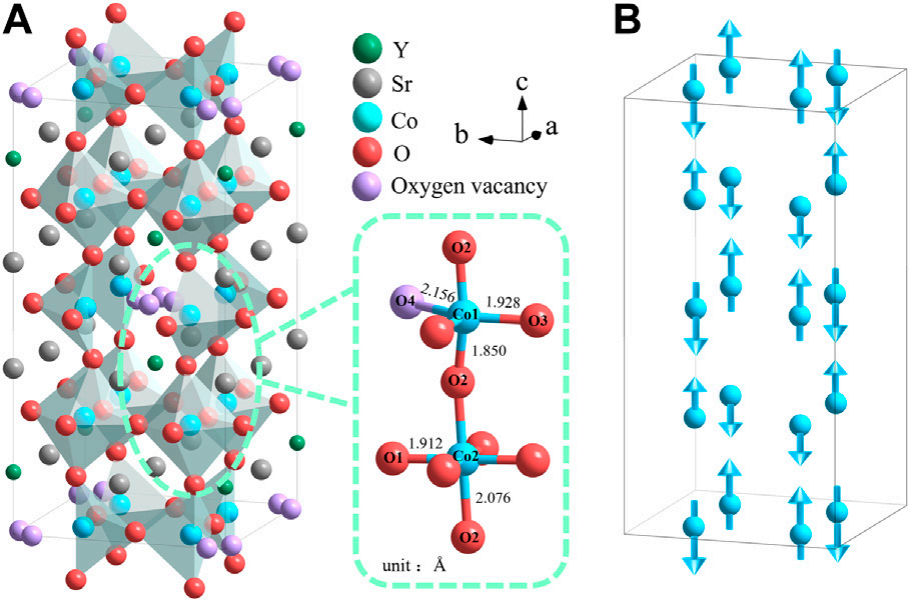
**(A)** Crystal structure of 314-SYCO shows the connection between the Co1O_4+1_ polyhedra and Co2O_6_ octahedra from neighbouring layers. **(B)** *G*-type antiferromagnetic ordering of Co cations along the *c*-axis.

The oxygen-deficient perovskite Sr_3_YCo_4_O_10.5+*δ*_ (314-SYCO) with an ordered tetragonal structure has potential applications in microwave absorbing materials, solid oxide fuel cells, and other fields owing to its room-temperature ferromagnetism (Golosova et al., 2009; Marik et al., 2018; Istomin et al., 2003), orbital and charge ordering (Khalyavin et al., 2011; Sheptyakov et al., 2009; Kishida et al., 2016), high electronic conductivity, and excellent activity for the oxygen-reduction reaction (Li et al., 2011; Lalan et al., 2019). The *A*-site ordered (AO) and oxygen vacancy ordered (OO) tetragonal superstructure of 314-SYCO is composed of alternating octahedral CoO_6_ layers and tetrahedral CoO_4.25+*δ*_ layers stacked along the *c*-axis. The *A*-site cations are arranged as –Sr–Y–Y–Sr– units along the *c*-axis, and the Sr^2+^: Y^3+^ ratio is 3:1 in the *ab*-plane. The oxygen vacancies of the CoO_4.25+*δ*_ layer are arranged in a zigzag pattern in the *bc*-plane (Khalyavin et al., 2011). For *δ* = −0.26–0.3, 314-SYCO usually has an AO/OO tetragonal superstructure, as illustrated in Figure 1A (Istomin et al., 2003; Fukushima et al., 2009). Moreover, the basic magnetic structure of 314-SYCO is a *G*-type antiferromagnet, as illustrated in Figure 1B (Sheptyakov et al., 2009). The magnetic properties are mainly derived from oxygen ordering at oxygen-vacancy sites (Kobayashi et al., 2005; Fukushima et al., 2008; Fukushima et al., 2009).

The 314-SYCO material has a complex and interesting phase-transition process. Hu et al. (Hu et al., 2017) observed an exothermic peak at 963°*C* in differential scanning calorimetry (DSC) curves, corresponding to the crystallisation of 314-SYCO. In addition, a weight loss at 400°*C* in the thermogravimetric (TG) curves of 314-SYCO samples in oxygen, air, and helium atmospheres were observed due to the removal of O4 (the Co1–O4 distance is the longest, as illustrated in Figure 1). At approximately 1,000°*C* in a helium atmosphere, the oxygen content in polycrystals that had lost most of their O4 was close to 10 (Sr_3_YCo_4_O_10_), resulting in a brownmillerite-type structure (Istomin et al., 2003). However, there are limited reports on the phase transition of 314-SYCO during its synthesis.

The phase structure, ordering, and physical properties of 314-SYCO could be modulated by Co-site doping. With increased Fe doping, Sr_0.75_Y_0.25_Co_1−*x*_Fe_*x*_O_2.625+*δ*_ changes from an ordered tetrahedral structure to a disordered cubic structure, while Sr_0.75_Y_0.25_Co_1−*x*_Ga_*x*_O_2.625+*δ*_ has a tetragonal superstructure with decreased magnetic order at *x* = 0.25 (Lindberg et al., 2006). In the CoO_6_ layer and/or antiferromagnetic CoO_4.25_ layer, Ga^3+^ ions both replace the high-spin-state Co^3+^ ions and increase the saturation magnetisation of Co ions. Further, it was shown that both the thermal expansion coefficient and conductivity of Sr_3_YCo_4−*x*_Fe_*x*_O_10.5+*y*_ decrease at 1173 K (Istomin et al., 2008). According to previous studies, intermediate-spin state Co^3+^ ions get preferentially replaced by Al^3+^ ions in the ferrimagnetic CoO_6_ layer, which lowers the Sr_3.1_Y_0.9_Co_4_O_10.5_ saturation magnetisation (Tsuruta et al., 2020). The addition of Al^3+^ disrupts the oxygen-ordering superstructure, which further inhibits the ferromagnetism of 314-SYCO at room temperature (Rajan and Subodh, 2020). As Cu has a similar ionic radius to Co, Cu doping at the Co site can directly increase carrier concentration. Therefore, the electrical properties of 314-SYCO can be significantly improved by substituting Co with Cu. In addition, CuO, as a sintering agent, increases the content of the liquid phase during sintering, thus improving the sintering quality. Our research group first proposed that Cu doping can reduce the resistivity of 314-SYCO (Du et al., 2014). However, a thorough investigation of the effects of Cu doping on the electromagnetic characteristics of polycrystalline 314-SYCO has not yet been conducted. The oxygen-deficient perovskite 314-SYCO has been proven to be useful as a microwave absorber or as a cathode for solid oxide fuel cells due to its high electronic conductivity and excellent activity for oxygen reduction reactions (Li et al., 2011; Lalan et al., 2019). Moreover, low electrical resistivity is an important physical parameter for microwave absorbers or cathodes for solid oxide fuel cells.

Here, the ordered phase transformation of 314-SYCO is reported for the first time. The microstructure, electrical transport, and magnetic properties of Sr_3_YCo_4−*x*_Cu_*x*_O_10.5+*δ*_ (*x* = 0, 0.2, 0.4) were investigated in detail. For *x* = 0–0.4, the ordered tetragonal Sr_3_YCo_4−*x*_Cu_*x*_O_10.5+*δ*_ phase was formed. The key step in the formation of the ordered tetragonal phase is an exothermic reaction at ∼1,042°*C* and an oxygen mass gain of ∼0.8%. With increasing Cu content, the electrical resistivity decreases greatly, and the polycrystalline material shows the three-dimensional Mott variable-range hopping mechanism typical of a semiconductor. Additionally, a *G*-type antiferromagnetic-to-ferromagnetic phase transition occurs due to a reduction in the spin state of some Co^3+^ ions.

## 2 Material and methods

### 2.1 Materials and synthesis

Using a solid-state reaction technique, Sr_3_YCo_4−*x*_Cu_*x*_O_10.5+*δ*_ (*x* = 0, 0.2, 0.4, 0.6, and 0.8) was made polycrystalline. The raw materials were SrCO_3_ (99.95%), Y_2_O_3_ (99.9%), Co_3_O_4_ (99.9%), and CuO (99%). All starting materials were purchased from Shanghai Aladdin Biochemical Technology Co., Ltd. (Shanghai, China). Stoichiometric amounts of the starting reagents were weighed (Sr: Y: Co: Cu = 3:1: 4−*x*: *x*) and homogeneously mixed in an agate mortar for at least 120 min. Following this, the powdered mixture was pressed into disc-shaped tablets (4 MPa/5 min +5 MPa/5 min) with a diameter of 20 mm and height of 3.0–3.5 mm, which were sintered in air at 1,100°*C* for 24 h.

### 2.2 Characterization of samples

The phase transitions during the sintering of the Sr_3_YCo_4−*x*_Cu_*x*_O_10.5+*δ*_ raw ingredients were investigated using TG-DSC (STA449F3, Netzsch, Selb, Germany) from 25 to 1,100°*C* at a heating rate of ∼10°C/min; the ingredient quantities were 7.5–9.5 mg. The phase structure of the sintered samples was identified using X-ray diffraction (XRD; Ultima IV, Rigaku Corporation, Tokyo, Japan; CuKα radiation with wavelength λ = 1.5406 Å, step size of 0.02°) through *θ–2θ* scans (40 kV, 40 mA, scan range of 10–100°, scan rate of 4°/min) and slow scans (40 kV, 40 mA, scan ranges of 20–21°, 38.5–39.5°, or 46.5–48°, scan rate of 0.4°/min). High-resolution transmission electron microscopy (HRTEM; Tecnai G2 F30 S-TWIN, FEI Company, Oregon, United States) and fast Fourier transform (FFT) analyses were used to establish the crystal structure. To examine the morphologies and Cu ion distributions, scanning electron microscopy coupled with energy-dispersive spectrometry (SEM-EDS; XL30ESEM, Philips, Amsterdam, Netherlands) was used. The sample density was measured using Archimedes’ method. X-ray photoemission spectroscopy (XPS) was conducted on an electron spectrometer (PHI5000 VersaProbe III, ULVAC-PHI, Inc., Kanagawa, Japan) with a monochromatic Al Kα irradiation source. Using a four-probe method, resistivity-temperature (*ρ-T*) curves were produced for the temperature range of 75–300 K. Seebeck coefficient*-*temperature (*S-T*) curves were recorded using a Seebeck measurement device (LSR-3/1,000, Linseis Messgeräte GmbH, Selb, Germany) in the temperature range of 300–1100 K. Using a superconducting quantum interference device (MPMS-XL, Quantum Design, Inc., California, United States) with a magnetic field of ∼1 T and a temperature range of 4–380 K, magnetisation*-*temperature (*M-T*) curves were measured.

## 3 Results and discussion

**FIGURE 2:**
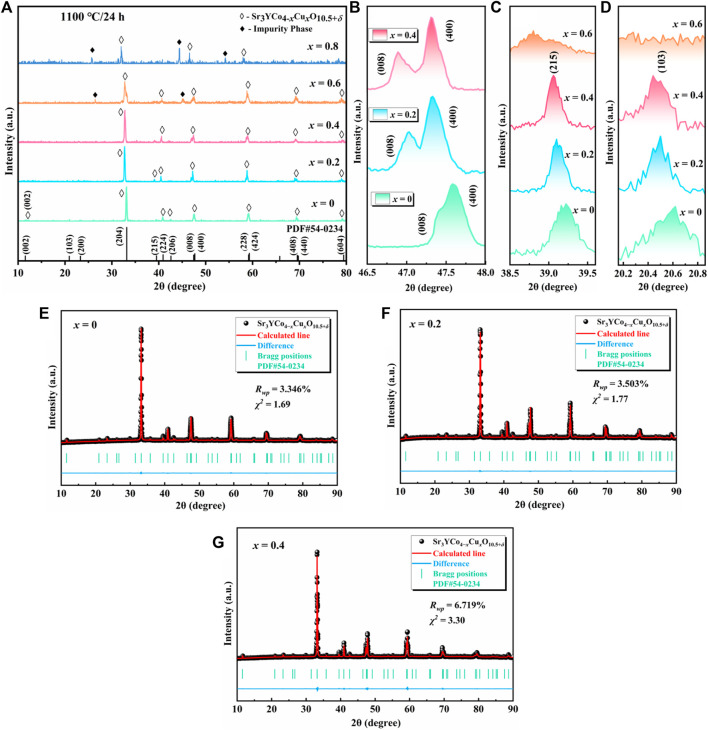
XRD patterns of Sr_3_YCo_4−*x*_Cu_*x*_O_10.5+*δ*_ (*x* = 0–0.8) polycrystals sintered at 1,100°*C* for 24 h **(A)** *x* = 0–0.8; slow scans (0.4°/min) of **(B)** (008)(400) split peak, **(C)** (103) peak, and **(D)** (215) peak. **(E–G)** XRD refinement results of Sr_3_YCo_4−*x*_Cu_*x*_O_10.5+*δ*_ (*x* = 0–0.4).

Figure 2 shows the XRD patterns of the polycrystalline Sr_3_YCo_4−*x*_Cu_*x*_O_10.5+*δ*_ (*x* = 0–0.8) sintered at 1,100°*C* for 24 h. For *x* = 0–0.4, all diffraction peaks were indexed to the tetragonal superstructure (PDF#54–0,234), i.e., *I4/mmm*. As the Cu doping content increased, the diffraction peaks of the samples shifted toward a lower angle, indicating an increase in the lattice constant due to the partial replacement of Co^3+/4+^ (0.61/0.53 Å) by Cu^2+^ (0.73 Å) (Hsieh and Fung, 2008; Zhao et al., 2010). Figures 2A,B show the presence of (008)(400), (228)(424), and (408)(440) split peaks. Both sets of spectra indicate *a* = *b* ≠ 1/2*c*, which is characteristic of a tetragonal structure (Hu et al., 2017). The peaks of (103) and (215) in Figures 2C,D imply that Sr_3_YCo_4−*x*_Cu_*x*_O_10.5+*δ*_ polycrystals had an ordered lattice, consistent with the ordered tetragonal phase extinction law (h, k, l ≠ 2n) (Kobayashi et al., 2005). Figures 2E–G show that the experimentally measured XRD diffraction peaks exhibit a high degree of overlap with the feasibility of Rietveld refinement, indicating that the samples from *x* = 0 to 0.4 are pure ordered tetragonal phase. The final reliability factors for the recorded patterns are *R*_*wp*_ = 3.346% and *χ* ^2^ = 1.69 for *x* = 0, *R*_*wp*_ = 3.503% and *χ* ^2^ = 1.77 for *x* = 0.2, and *R*_*wp*_ = 6.719% and *χ* ^2^ = 3.30 for *x* = 0.4.

The average grain size in the direction of the vertical grain plane (204), *D*_(204)_, was calculated using Scherrer equation $D_{D_{\left(204\right)}}=K\lambda /\beta \cos\theta$ (Vinila and Isac, 2022). Here, *β* is the full width at half maximum of the peak, and *K* = 0.89. Table 1 summarises the lattice constant, grain size, linear shrinkage, and density of Sr_3_YCo_4−*x*_Cu_*x*_O_10.5+*δ*_ (*x* = 0, 0.2, and 0.4) samples. The linear shrinkage (Δ*L*/*L*_0_, where Δ*L* is the shortening value after sintering and *L*_0_ is the diameter of the sample before sintering) and bulk density (*P*) significantly increased with increasing *x*. In Figures 2A,C,D, the (103) and (215) diffraction peaks are very small for *x* = 0.6–0.8, suggesting that the ordered phase was either minimal or not present.

**TABLE 1:** Crystal and physical properties of Sr_3_YCo_4−*x*_Cu_*x*_O_10.5+*δ*_ polycrystals sintered at 1,100°*C* for 24 h.

Sample	Lattice constant	Grain size	Linear shrinkage	Density	
	*a, b* (Å)	*c* (Å)	*D*_(204)_ (nm)	Δ*L*/*L*_0_ (%)	*P (*g/cm^3^ *)*
*x* = 0	7.63	15.35	48.8	15.6	3.82
*x* = 0.2	7.67	15.41	54.5	20.5	4.90
*x* = 0 .4	7.70	15.43	59.4	22.1	5.08

**FIGURE 3:**
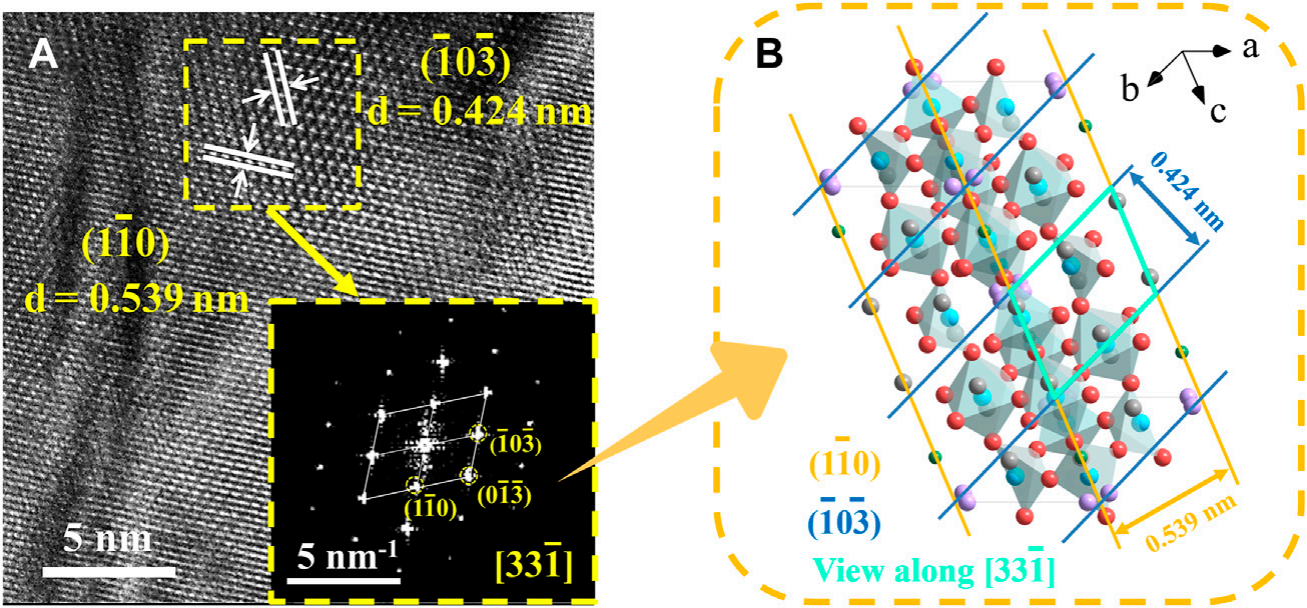
**(A)** HRTEM image of Sr_3_YCo_4−*x*_Cu_*x*_O_10.5+*δ*_ polycrystals: *x* = 0.2 (ordered tetrahedral). The inset shows the corresponding FFT pattern of Sr_3_YCo_4−*x*_Cu_*x*_O_10.5+*δ*_. **(B)** Relationship between the FFT diffraction spots and the corresponding crystal structure.

Figure 3A displays the HRTEM pictures together with their accompanying FFT diffraction spots, and Figure 3B illustrates how the crystal structure of the Sr_3_YCo_4−*x*_Cu_*x*_O_10.5+*δ*_ (*x* = 0.2) samples correspond to the diffraction spots. The *d*-spacings of 0.539 and 0.424 nm are indexed to the $\left(1\bar{1}0\right)$ and $\left(\bar{1}0\bar{3}\right)$ lattice planes, respectively. The FFT pattern (inset in Figure 3A) and Figure 3B show the diffraction spots corresponding to the $\left(1\bar{1}0\right)$ , $\left(0\bar{1}\bar{3}\right)$ , and $\left(\bar{1}0\bar{3}\right)$ planes along the $\left[33\bar{1}\right]$ orientation. The superlattice (103) diffraction surface confirms that the tetragonal Sr_3_YCo_4−*x*_Cu_*x*_O_10.5+*δ*_ polycrystalline structure has an ordered superstructure.

**FIGURE 4:**
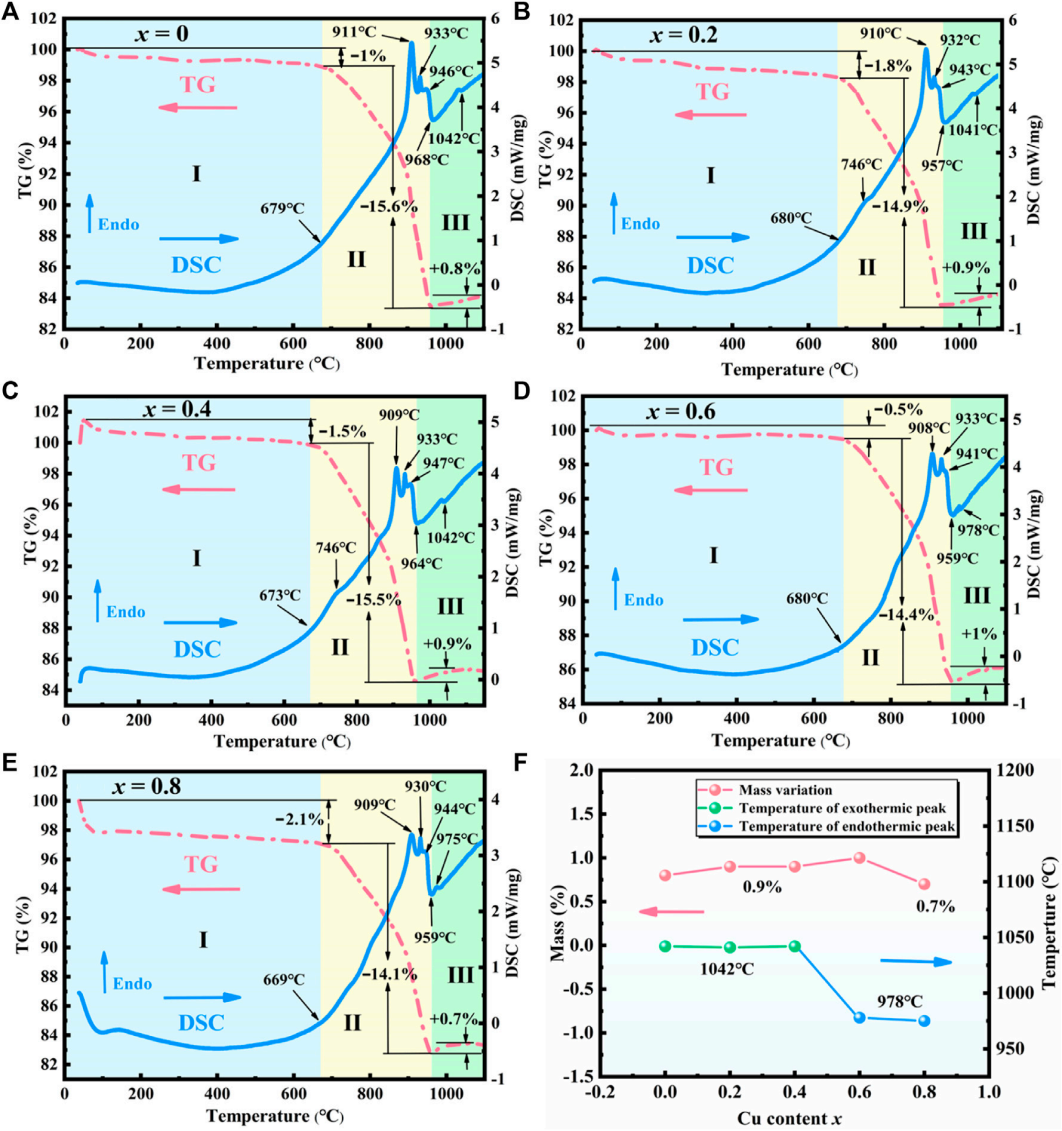
TG*-*DSC curves of the sintering ingredients of Sr_3_YCo_4−*x*_Cu_*x*_O_10.5+*δ*_ for *x* values of **(A)** 0, **(B)** 0.2, **(C)** 0.4, **(D)** 0.6, **(E)** 0.8. **(F)** Mass variation and thermal effects for *x* = 0–0.8 in stage Ⅲ.

TG-DSC curves were measured to simulate the sintering of the raw ingredients to produce Sr_3_YCo_4−*x*_Cu_*x*_O_10.5+*δ*_ (*x* = 0–0.8). Figures 4A–E show three stages in all TG curves, i.e., approximately 25–680°*C* for stage I, 680–969°*C* for stage II, and 969–1,100°*C* for stage III. The curves were similar for all samples in stages I and II.

In stage Ⅰ, the mass loss of all samples was in the range of 0.5–2.5% due to the dehydration of the raw materials. The mass loss was in the range of 12–15.6% in stage II. The endothermic peak at ∼910°*C* corresponds to the phase transition from orthorhombic to trigonal SrCO_3_ and the decomposition of Co_3_O_4_ into CoO and O_2_ (Delorme et al., 2015). Trigonal SrCO_3_ is broken into SrO and CO_2_, which produce the endothermic peak at ∼933°*C* (Ptáček et al., 2015).

As the temperature increased above 946°*C*, all TG curves showed further weight loss, indicating that SrCO_3_ continued to decompose. In the DSC curves, the exothermic peak at ∼968°*C* corresponds to the crystallisation of Sr_3_YCo_4–*x*_Cu_*x*_O_10.5_, because one of the four adjacent oxygen vacancies (Figure 1A) can be occupied easily (O_10_→O_10.5_) (Istomin et al., 2003; Rupasov et al., 2009), as indicated by Eq. 1.

$$\left(4–x\right)CoO+3SrO+\frac{1}{2}Y_{2}O_{3}+xCuO+O_{2}\;\overset{968^{{}^{\circ}}C}{\to }{\;Tetragonal-Sr}_{3}Y{Co}_{4–x}{Cu}_{x}O_{10.5}$$ (1)

For *x* = 0.2–0.8, the weak endothermic peak observed at 746–811°*C* may be related to a trace eutectic mixture formed by CuO and SrCO_3_–Y_2_O_3_–Co_3_O_4_ (Kingery and Narasimhan, 1959).

In stage Ⅲ, the mass variation and thermal behaviour are significantly dependent on *x*. As shown in Figure 4F, the mass gain is 0.8–0.9% for *x* = 0–0.4, while the DSC curves show a weak exothermic peak at approximately 1,042°*C*, corresponding to the uptake of oxygen (*δ*) (Eq. 2).

$$Tetragonal-{Sr}_{3}Y{Co}_{4–x}{Cu}_{x}O_{10.5}+\frac{\delta }{2}O_{2}\overset{1042^{{}^{\circ}}C}{\to }\;Ordered\;tetragonal{-Sr}_{3}Y{Co}_{4–x}{Cu}_{x}O_{10.5+\delta }$$ (2)

For *x* = 0.6–0.8, the weak endothermic peak at 978°*C* may correspond to an impurity formed by heating. Despite a mass gain of 0.7–1%, the exothermic peaks related to the ordered phase were not observed at ∼1,042°*C*. This observation is consistent with the XRD patterns in Figure 2, where the (103) and (215) peaks were not observed for *x* = 0.6. The lack of the ordered phase may be caused by excessive Cu doping, implying that the solid solubility of Cu is *x* = 0.4–0.6.

**FIGURE 5:**
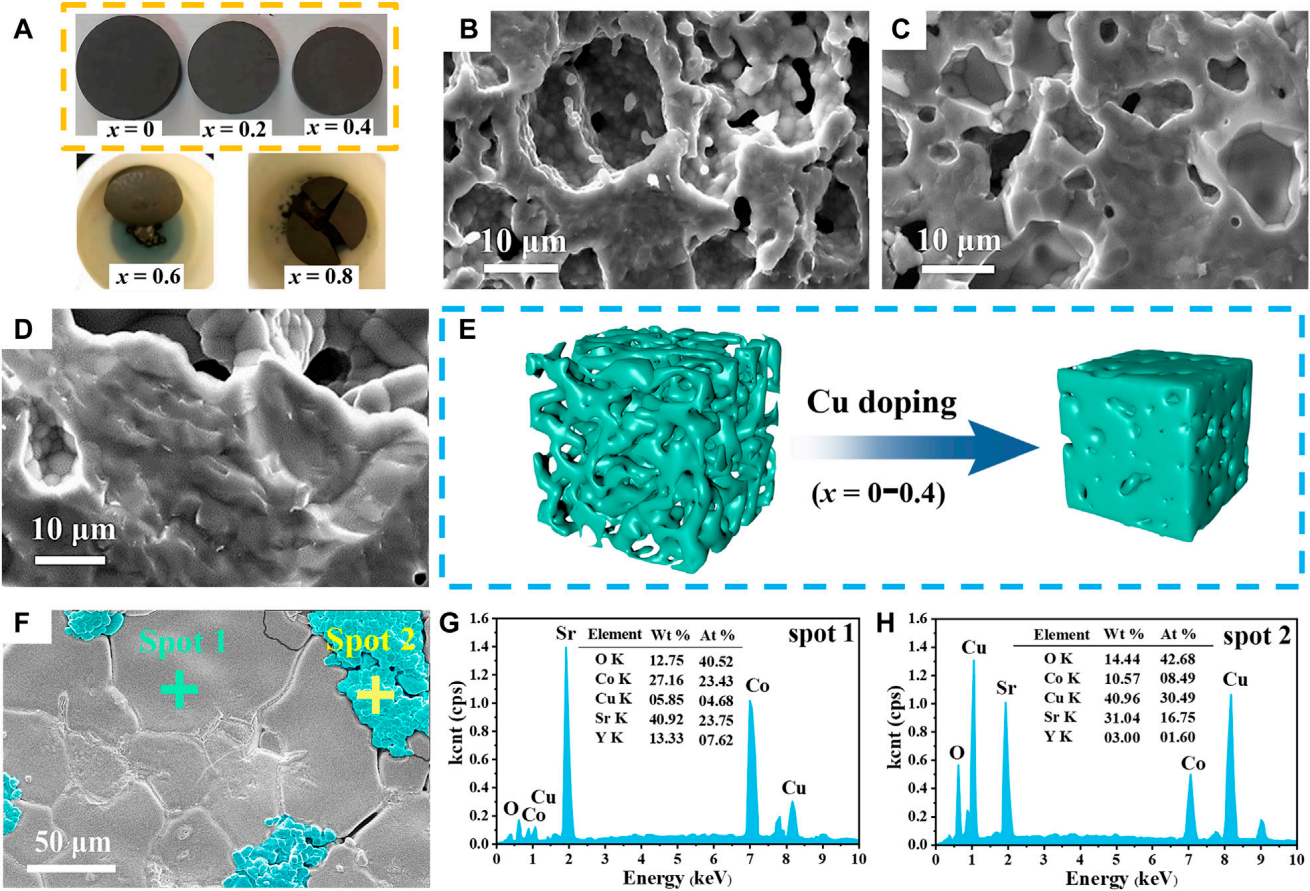
**(A)** Photographs of Sr_3_YCo_4−*x*_Cu_*x*_O_10.5+*δ*_ samples sintered at 1,100°*C* for 24 h for *x* values of 0, 0.2, and 0.4, 0.6, and 0.8. Cross-sectional SEM images of Sr_3_YCo_4−*x*_Cu_*x*_O_10.5+*δ*_ for *x* values of: **(B)** 0, **(C)** 0.2, and **(D)** 0.4. **(E)** Schematic of the morphological changes (*x* = 0–0.4). **(F)** Surface SEM image of the Sr_3_YCo_4−*x*_Cu_*x*_O_10.5+*δ*_ polycrystal (*x* = 0.6). Sample *x* = 0.6 EDS spectra at **(G)** spot 1 and **(H)** spot 2 in **(F)**.

Figure 5A shows photographs of polycrystalline Sr_3_YCo_4−*x*_Cu_*x*_O_10.5+*δ*_ sintered at 1,100°*C* for 24 h. For *x* = 0–0.4, the surfaces are smooth and flat, and the volume drastically reduced with increasing *x* (i.e., enhanced sintering). For *x* = 0.6–0.8, the corrosion of the samples with the crucible became increasingly severe as the dissolution exceeded the solid limit, and low-melting-point heterogeneous phases were produced. Cross-sectional SEM images of polycrystalline Sr_3_YCo_4−*x*_Cu_*x*_O_10.5+*δ*_ (*x* = 0–0.4) (Figures 5B–D) indicate the porous structures of these samples. With increasing *x*, an increased density is observed as a result of sintering, i.e., the pore volume reduced to form close grain connections (Figure 5E). These trends also align with the findings in Table 1. Figure 5F shows a surface SEM image of Sr_3_YCo_4−*x*_Cu_*x*_O_10.5+*δ*_ (*x* = 0.6) polycrystalline material. The grain clusters are highlighted in blue and the lighter lines are the grain boundaries. According to EDS analysis, spot 2 (Figure 5H) has significantly higher Cu content than spot 1 (Figure 5G). Therefore, the grain clusters are thought to be a Cu-rich phase.

**FIGURE 6:**
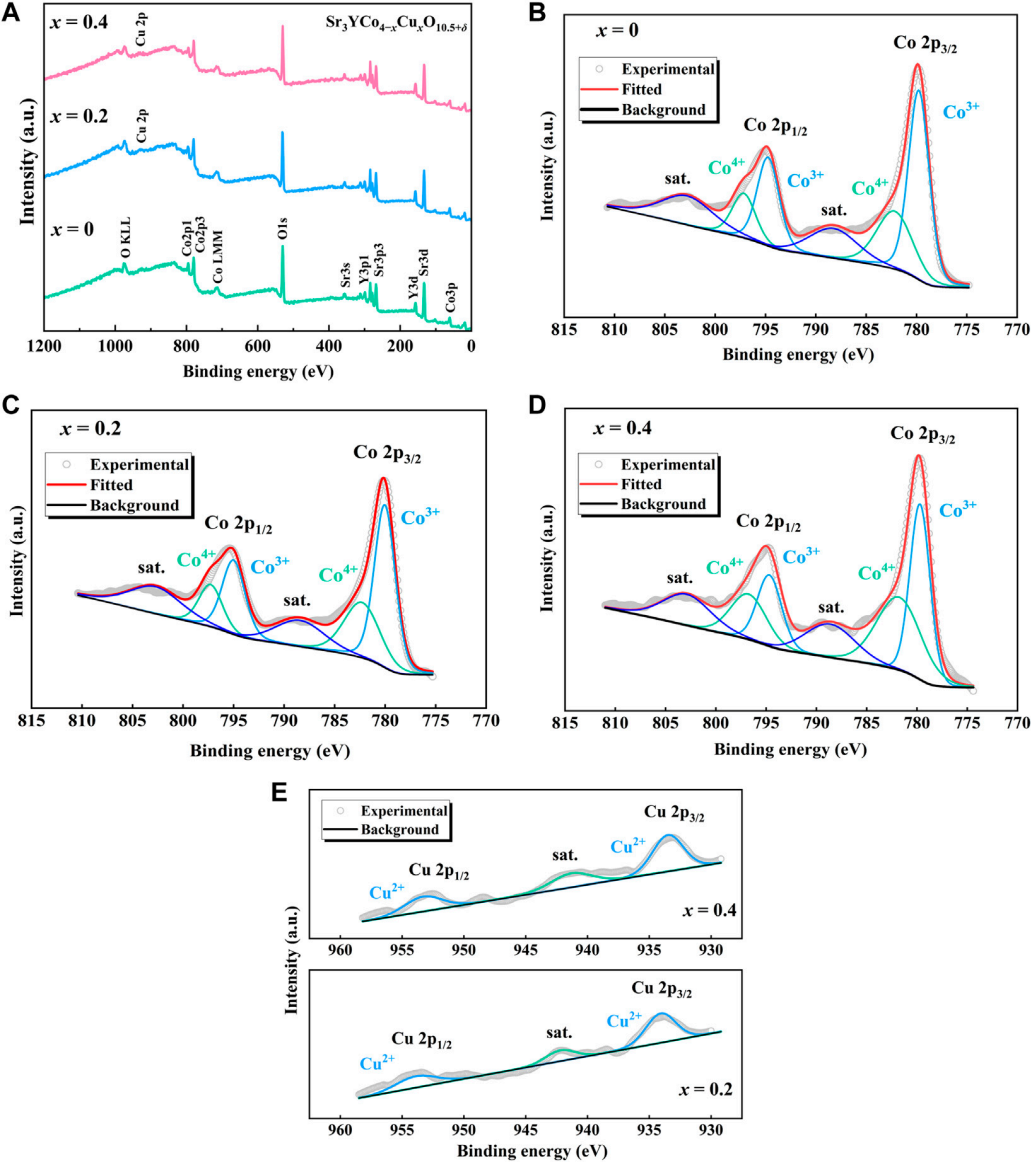
**(A)** Survey spectra of Sr_3_YCo_4−*x*_Cu_*x*_O_10.5+*δ*_ samples (*x* = 0, 0.2, and 0.4). **(B–D)** Co 2p XPS spectra of Sr_3_YCo_4−*x*_Cu_*x*_O_10.5+*δ*_ samples (*x* = 0, 0.2, and 0.4). **(D)** Cu 2p XPS spectra of Sr_3_YCo_4−*x*_Cu_*x*_O_10.5+*δ*_ samples (*x* = 0.2 and 0.4).

The XPS survey spectra of Sr_3_YCo_4−*x*_Cu_*x*_O_10.5+*δ*_ samples (*x* = 0, 0.2, and 0.4) are shown in Figure 6A, confirming the presence of elements in these samples; no other impurity elements were detected, demonstrating the high purity of the experimentally-produced polycrystals. The XPS Co elemental spectra for the samples (*x* = 0, 0.2, and 0.4) are presented in Figures 6B–D. In contrast with the Co^3+^ ion, Co^4+^ contains a more positive electrical charge and lower electron cloud density, thus the binding energy of 2p electrons should also be higher (LU et al., 2013). As a result, Co^3+^ ions have a lower binding energy than Co^4+^ ions. Therefore, the intensity peaks appearing at 779.77/794.74 eV for *x* = 0, 780.5/795.02 eV for *x* = 0.2 and 779.68/794.65 eV for *x* = 0.4 can be assigned to Co^3+^, while the peaks of 782.18/797.15 eV for *x* = 0, 782.36/797.33 eV for *x* = 0.2 and 781.81/796.78 eV for *x* = 0.4 can be assigned to Co^4+^ ion. For each composition, the Co^3+^/Co^4+^ ratio is determined by fitting the area under the curve. The Co^3+^/Co^4+^ ratio for each composition is presented in Table 2. It was observed that the Co^3+^ content decreased significantly with the increase in the Cu doping amount. The XPS results confirm that the Cu^2+^ ions predominantly occupy Co^3+^ sites. In Figure 6E, the Cu^2+^ signal (Cu2p_3/2_ and Cu2p_1/2_ are 934.07 and 953.87 eV for *x* = 0.2, 933.49 and 953.29 eV for *x* = 0.4, respectively) and its satellite peak (936.0–946.0 eV) in the Cu 2p spectral range are obvious (Wu et al., 2018; Li et al., 2022). The results show that in Sr_3_YCo_4−*x*_Cu_*x*_O_10.5+*δ*_, Cu ions exist in the oxidation state of +2 valence. Compared to the element Co, Cu 2p is relatively weak. This phenomenon is consistent with its low element content.

**TABLE 2:** Co^3+^/Co^4+^ ion ratio in Sr_3_YCo_4−*x*_Cu_*x*_O_10.5+*δ*_ (*x* = 0, 0.2, and 0.4).

Compound	Sr_3_YCo_4_O_10.5+*δ*_	Sr_3_YCo_3.8_Cu_0.2_O_10.5+*δ*_	Sr_3_YCo_3.6_Cu_0.4_O_10.5+*δ*_
Co^3+^/Co^4+^ ratio	1.877	1.633	1.141

**FIGURE 7:**
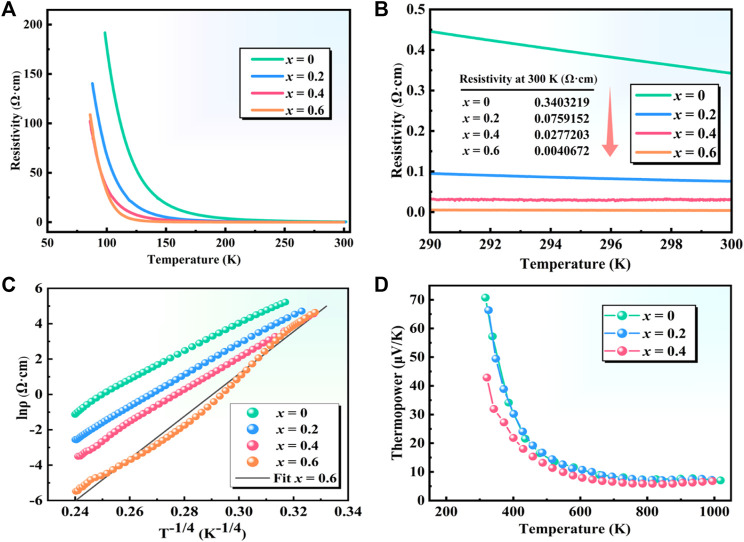
*ρ-T* curves of polycrystalline Sr_3_YCo_4*−x*_Cu_*x*_O_10.5+*δ*_ (*x* = 0*–*0.6): **(A)** 50*–*300 K, **(B)** 290*–*300 K. This is a Kelvin temperature unit. **(C)** *lnρ-T* ^*−*1/4^ and **(D)** *S-T* curves of polycrystalline Sr_3_YCo_4−*x*_Cu_*x*_O_10.5+*δ*_ (*x* = 0*–*0.4).

As shown in Figure 7A, the resistivity-temperature (*ρ-T*) curves of polycrystalline Sr_3_YCo_4−*x*_Cu_*x*_O_10.5+*δ*_ (*x* = 0–0.6) showed electrical transport characteristics typical of a semiconductor. The resistivity decreased significantly from *x* = 0 to 0.4 and stabilised from *x* = 0.4 to 0.6. Figure 7B shows that the room temperature resistivity at 300 K for *x* = 0.4 and *x* = 0.6 is 0.0277203 Ω cm and 0.0040672 Ω cm, respectively. Compared with the room temperature resistivity at *x* = 0 of 0.3403219 Ω cm, the resistivity decreased by 92–99% with Cu doping.

The *lnρ-T* ^−1/(n+1)^ curve was fitted using the Mott variable-range hopping model expressed in Eq. 3 (Mott, 1969):

$$\rho =\rho_{0\cdot }\exp{\left(T_{m}/T\right)}^{1/\left(n+1\right)}$$ (3)

Here, *ρ*_0_ is the initial electrical resistivity (constant), *T*_*m*_ is the characteristic temperature of the variable-range hopping mechanism, and *n* = 2 or *n* = 3 is the dimension of the system. Figure 7C shows that when *n* = 3, *lnρ-T* ^*−*1/4^ is almost linear for *x* = 0–0.04, indicating that a small number of Co^4+^ ions provide hole carriers (Terasaki et al., 2010), and hopping conduction occurs between Co^4+^ ions *via* a three-dimensional variable-range hopping mechanism. A deviation was found between the experimental and fitted curves for the *x* = 0.6 sample, which may be caused by changes in the resistivity induced by impurity phases produced beyond the limit of solid solubility of Cu in the perovskite structure. When Co^3+/4+^ was replaced with Cu^2+^, the concentration of hole carriers increased. In addition, the mobility of the hole carriers increased as the grain size and density of Sr_3_YCo_4*−x*_Cu_*x*_O_10.5+*δ*_ increased due to sintering.

The *S-T* curves of Sr_3_YCo_4−*x*_Cu_*x*_O_10.5+*δ*_ (*x* = 0*–*0.4) in Figure 7D indicate that the thermopower decreased with increasing temperature. The curves for *x* = 0 and 0.2 are very similar, and the thermopower of the *x* = 0.4 material was significantly lower due to the higher carrier concentration. In contrast, increasing the spin entropy of the Co^3+^ ions increases the thermopower (Yoshida et al., 2009).

**FIGURE 8:**
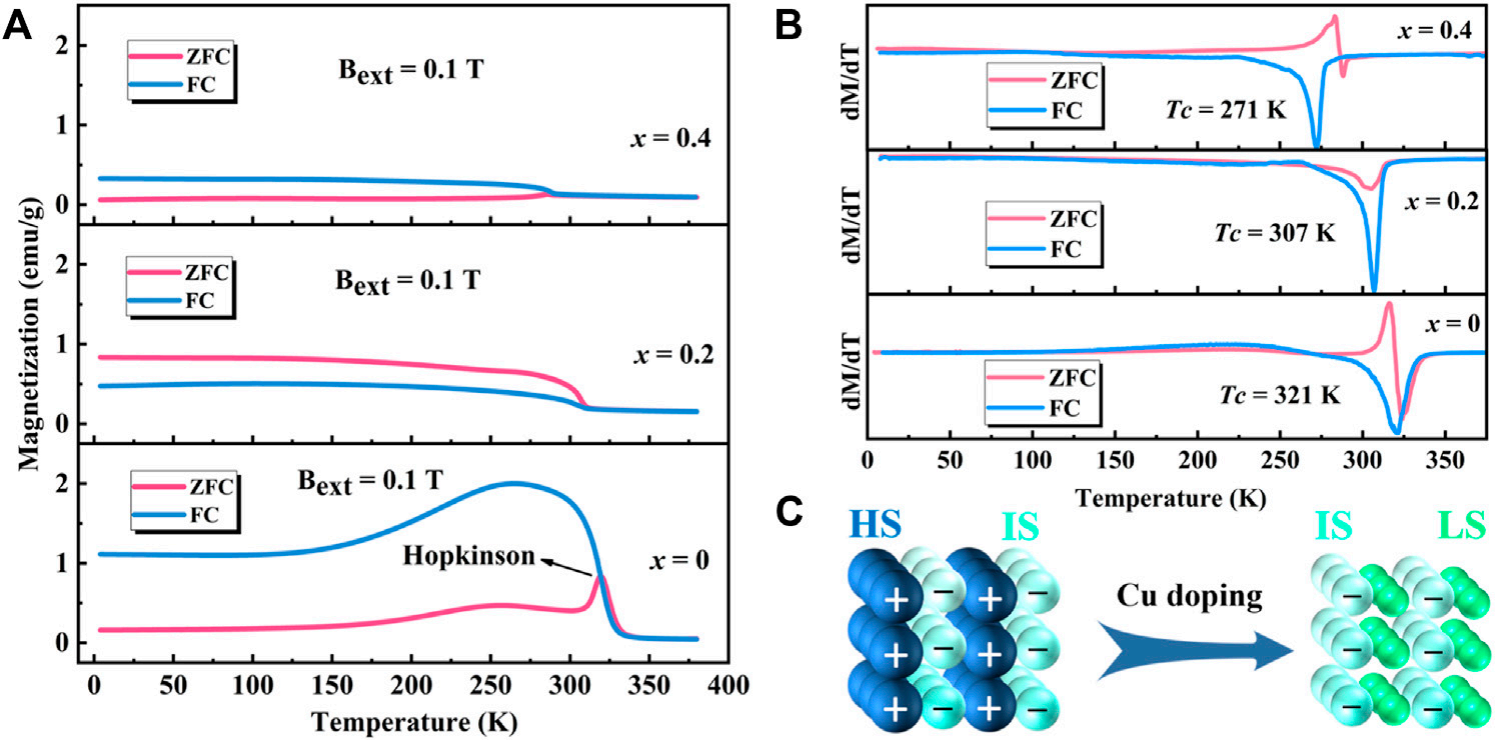
Magnetic properties of Sr_3_YCo_4−*x*_Cu_*x*_O_10.5+*δ*_ (*x* = 0*–*0.4): **(A)** *M-T,* **(B)** *dM/dT-T* curves, and **(C)** spin states of Co^3+^ ions in Sr_3_YCo_4*–x*_Cu_*x*_O_10.5+*δ*_.

The *M-T* and *dM/dT-T* curves of polycrystalline Sr_3_YCo_4−*x*_Cu_*x*_O_10.5+*δ*_ (*x* = 0–0.4) are shown in Figures 8A,B. For *x* = 0, the Hopkinson peak is observed at 319 K in the zero-field-cooling (ZFC) curve. Magnetic moments in the magnetic domains of materials are randomly distributed and frozen at low temperatures, and the net magnetic moments tend to be zero in *G*-type antiferromagnetic phases (Sheptyakov et al., 2009; Troyanchuk et al., 2009; Troyanchuk et al., 2019). As the temperature increases, the magnetic moments rotate to align along the direction of the external magnetic field, while the magnetic domain wall moves and the magnetic domain grows. When all magnetic moments are aligned in the same direction, the magnetic domain walls disappear, and the magnetisation reaches its maximum at 319 K. At *T_c_*, thermal agitation causes magnetic moments to misalign, resulting in a ferromagnetic-paramagnetic second-order transition and a decrease in magnetisation. There is no resistance between the magnetic domain walls when the field-cooling (FC) curve is recorded, and the magnetic moments are aligned in the same direction under an external magnetic field. Therefore, the maximum magnetisation obtained for the FC curve was higher than that obtained for the ZFC curve. The FC curves had the highest peak at 267 K. As the temperature continues to decrease, a ferromagnetic-antiferromagnetic transition occurs, and a *G*-type antiferromagnetic phase is generated (Lindberg et al., 2006). The separation of the ZFC and FC curves at low temperature indicates that spin-glass state-like components may be present (Motohashi et al., 2005; Sutjahja et al., 2015; Guo et al., 2018; Srivastava et al., 2020).

For *x* = 0.2, the maximum magnetisation of the ZFC curve was found at 4 K, attributed to the chemical compressive stress induced by lattice distortion due to Cu doping. It allows most Co^3+^ ions to transit from a high/intermediate spin (HS/IS, *t*_*2g*_^4^ *e*_*g*_^2^/*t*_*2g*_^5^ *e*_*g*_^1^, *S* = 2/1) to an intermediate/low spin (IS/LS, *t*_*2g*_^5^ *e*_*g*_^1^/*t*_*2g*_^6^ *e*_*g*_^0^, *S* = 1/0), resulting in a decrease in magnetisation (Tsuruta et al., 2020). The Co^3+^ ions in the LS state are nonmagnetic. At this point, the spin magnetic moments of the Co^3+^ ions in most regions are aligned in the same direction, as shown in Figure 8C, which is similar to the ferromagnetic structure. The *T_c_* from the ZFC curves decreased from 320 K (*x* = 0) to 307 K (*x* = 0.2) and 288 K (*x* = 0.4), which is related to the decrease in the spin state of Co^3+^ ions, leading to a decrease in magnetisation and weakening of the magnetic interaction. Moreover, in conjunction with the XPS results, the doping of Cu ions instead of Co ions led to a reduction in Co^3+^ ion content, which has a direct effect on the reduction of the magnetisation intensity of the sample.

## 4 Conclusion

A conventional solid-state reaction method was used to synthesise polycrystalline Sr_3_YCo_4−*x*_Cu_*x*_O_10.5+*δ*_ (*x* = 0–0.8). An ordered tetragonal phase and ordering phase transformations due to oxygen uptake above 1,000°*C* were observed for *x* = 0–0.4 for the first time. An ordered tetragonal phase and ordering phase transformation due to oxygen uptake above 1,000°*C* were observed for *x* = 0–0.4 for the first time. A study of the phase-transformations process during heating provides a research reference for optimising the conditions of the material synthesis process, such as sintering temperature and holding time. Cu doping was observed to significantly reduce the electrical resistivity, and the three-dimensional Mott variable-range hopping conduction mechanism of polycrystalline Sr_3_YCo_4−*x*_Cu_*x*_O_10.5+*δ*_ was explored. We also found that Cu doping reduced both the spin state and content of Co^3+^ ions, thereby inhibiting the room temperature ferromagnetism of 314-SYCO and providing a reference for doping to modulate the properties of oxygen-deficient perovskites.

## Data Availability

The original contributions presented in the study are included in the article/supplementary material, further inquiries can be directed to the corresponding author.

## References

[B1] Delorme F. Diaz-Chao P. Guilmeau E. Giovannelli F. (2015). Thermoelectric properties of Ca_3_Co_4_O_9_–Co_3_O_4_ composites. Ceram. Int. 41 (8), 10038–10043. 10.1016/j.ceramint.2015.04.091

[B2] Du X. L. Yu L. Zhang B. Song S. J. Li G. F. (2014). Effects of Cu doping on the microstructure and electrical properties of Sr_3_YCo_4_O_10.5+δ_ polycrystalline. Adv. Mat. Res. 934, 80–85. 10.4028/www.scientific.net/AMR.934.80

[B3] Fukushima S. Sato T. Akahoshi D. Kuwahara H. (2009). Order-disorder effect of A-site and oxygen-vacancy on magnetic and transport properties of Y_1/4_Sr_3/4_CoO_3−δ_ . J. Phys. Soc. Jpn. 78, 064706–6. 10.1143/JPSJ.78.064706

[B4] Fukushima S. Sato T. Akahoshi D. Kuwahara H. (2008). Comparative study of ordered and disordered Y_1−x_Sr_x_CoO_3−δ_ . J. Appl. Phys. 103, 07F705–4. 10.1063/1.2830615

[B5] Golosova N. O. Kozlenko D. P. Dubrovinsky L. S. Drozhzhin O. A. Istomin S. Y. Savenko B. N. (2009). Spin state and magnetic transformations in Sr_0.7_Y_0.3_CoO_2.62_ at high pressures. Phys. Rev. B 79, 104431–104437. 10.1103/PhysRevB.79.104431

[B6] Guo X. Tong P. Lin J. Yang C. Zhang K. Lin S. (2018). Effects of Cr substitution on negative thermal expansion and magnetic properties of antiperovskite Ga_1−x_Cr_x_N_0.83_Mn_3_ compounds. Front. Chem. 6, 75. 10.3389/fchem.2018.00075 29619367 PMC5871658

[B7] Hsieh C. Y. Fung K. Z. (2008). Effect of divalent dopants on defect structure and electrical properties of Bi_2_WO_6_ . J. Phys. Chem. Solids 69, 302–306. 10.1016/j.jpcs.2007.07.106

[B8] Hu J. L. Yu L. Du X. L. Song S. J. (2017). Preparation and properties of room-temperature ferromagnet Sr_3_YCo_4_O_10.5+δ_ polycrystals. J. Synth. Cryst. 46, 238–242. (Chinese). 10.3969/j.issn.1000-985X.2017.02.008

[B9] Istomin S. Y. Drozhzhin O. A. Napolsky P. S. Putilin S. N. Gippius A. A. Antipov E. V. (2008). Thermal expansion behavior and high-temperature transport properties of Sr_3_YCo_4−x_Fe_x_O_10.5+y_ *, x*=0.0, 1.0, 2.0 and 3.0. Solid State Ionics 179, 1054–1057. 10.1016/j.ssi.2008.01.017

[B10] Istomin S. Y. Grins J. Svensson G. Drozhzhin O. A. Kozhevnikov V. L. Antipov E. V. (2003). Crystal structure of the novel complex cobalt oxide Sr_0.7_Y_0.3_CoO_2.62_ . Chem. Mat. 15, 4012–4020. 10.1021/cm034263e

[B11] Khalyavin D. D. Chapon L. C. Suard E. Parker J. E. Thompson S. P. Yaremchenko A. A. (2011). Complex room-temperature ferrimagnetism induced by zigzag stripes of oxygen vacancies in Sr_3_YCo_4_O_10+δ_ . Phys. Rev. B 83, 140403–140411. 10.1103/PhysRevB.83.140403

[B12] Kingery W. D. Narasimhan M. D. (1959). Densification during sintering in the presence of a liquid phase. II. Experimental. J. Appl. Phys. 30, 307–310. 10.1063/1.1735156

[B13] Kishida T. Kapetanakis M. D. Yan J. Q. Sales B. C. Pantelides S. T. Pennycook S. J. (2016). The origin of magnetic ordering in Sr_3_YCo_4_O_10+x_ . Microsc. Microanal. 22, 1394–1395. 10.1017/S1431927616007819 PMC4730147 26818899

[B14] Kobayashi W. Ishiwata S. Terasaki I. Takano M. Grigoraviciute I. Yamauchi H. (2005). Room-temperature ferromagnetism in Sr_1−x_ *Y* _x_CoO_3−δ_ (0.2≤*x*≤0.25). Phys. Rev. B 72, 1–5. 10.1103/PhysRevB.72.104408

[B15] Lalan V. Puthiyedath Narayanan A. Surendran K. P. Ganesanpotti S. (2019). Room-temperature ferromagnetic Sr_3_YCo_4_O_10+δ_ and carbon black-reinforced polyvinylidenefluoride composites toward high-performance electromagnetic interference shielding. ACS Omega 4, 8196–8206. 10.1021/acsomega.9b00454 31459908 PMC6648688

[B16] Li W. Liang K. Wang J. Wen J. Shi J. Zhang Z. (2022). Effects of Cu doping on electrochemical NOx removal by La_0.8_Sr_0.2_MnO_3_ perovskites. Environ. Res. 210, 112955. 10.1016/j.envres.2022.112955 35182592

[B17] Li Y. Kim Y. N. Cheng J. Alonso J. A. Hu Z. Chin Y. Y. (2011). Oxygen-deficient perovskite Sr_0.7_Y_0.3_CoO_2. 65−δ_ as a cathode for intermediate-temperature solid oxide fuel cells. Chem. Mat. 23, 5037–5044. 10.1021/cm202542q

[B18] Lindberg F. Drozhzhin O. A. Istomin S. Y. Svensson G. Kaynak F. B. Svedlindh P. (2006). Synthesis and characterization of Sr_0.75_Y_0.25_Co_1−x_ *M* _x_O_2.625+δ_ (M=Ga, 0.125≤*x*≤0.500 and M=Fe, 0.125≤*x*≤0.875). J. Solid State Chem. 179, 1434–1444. 10.1016/j.jssc.2006.01.057

[B19] Lu Y. Chen L. Lu C. Ni Y. Xu Z. (2013). Effects of oxygen defects on structure and properties of Sm_0.5_Sr_0.5_CoO_3-δ_ annealed in different atmospheres. J. Rare Earths 31, 1183–1190. 10.1016/s1002-0721(12)60424-4

[B20] Marik S. Mohanty P. Singh D. Singh R. P. (2018). Moderate magnetic field induced large exchange bias effect in ferrimagnetic 314—Sr_3_YCo_4_O_10.5_ material. J. Phys. D. Appl. Phys. 51 (6), 065006. 10.1088/1361-6463/aaa452

[B21] Motohashi T. Caignaert V. Pralong V. Hervieu M. Maignan A. Raveau B. (2005). Competition between ferromagnetism and spin glass: The key for large magnetoresistance in oxygen-deficient perovskitesSrCo_1−x_ *Mx*O_3−δ_(M=Nb, Ru). Phys. Rev. B 71, 214424. 10.1103/physrevb.71.214424

[B22] Mott N. F. (1969). Conduction in non-crystalline materials: III. Localized states in a pseudogap and near extremities of conduction and valence bands. Philos. Mag. 19, 835–852. 10.1080/14786436908216338

[B23] Ptáček P. Bartoníčková E. Švec J. Opravil T. Šoukal F. Frajkorová F. (2015). The kinetics and mechanism of thermal decomposition of SrCO3 polymorphs. Ceram. Int. 41 (1), 115–126. 10.1016/j.ceramint.2014.08.043

[B24] Rajan A. Subodh G. (2020). Crystal structure, microstructure, and broadband electromagnetic response of Al^3+^-substituted Sr_3_YCo_4_O_10+δ_ double perovskites. Ceram. Int. 46 (16), 25683–25690. 10.1016/j.ceramint.2020.07.044

[B25] Rupasov D. Chroneos A. Parfitt D. Kilner A. Grimes W. Istomin S.-Y. (2009). Oxygen diffusion in Sr_0.75_Y_0.25_CoO_2.625_: A molecular dynamics study. Phys. Rev. B 79, 172102–172109. 10.1103/PhysRevB.79.172102

[B26] Sheptyakov D. V. Pomjakushin V. Y. Drozhzhin O. A. Istomin S. Y. Antipov E. V. Bobrikov I. A. (2009). Correlation of chemical coordination and magnetic ordering in Sr_3_YCo_4_O_10.5+δ_ (*δ*=0.02 and 0.26). Phys. Rev. B 80, 024409. 10.1103/PhysRevB.80.024409

[B27] Srivastava A. Singh A. K. Srivastava O. N. Tewari H. S. Masood K. B. Singh J. (2020). Magnetic and dielectric properties of La and Ni Co-substituted BiFeO_3_ nanoceramics. Front. Phys. 8. 10.3389/fphy.2020.00282

[B28] Sutjahja I. M. Berthalita F. Mustaqima M. Nugroho A. A. Tjia M. O. (2015). Effects of partial Co replacement by Fe in Sr_0.775_Y_0.225_CoO_3-δ_ on its magnetic property, oxygen deficiency and crystal structure. Mater. Pol. 33, 579–587. 10.1515/msp-2015-0078

[B29] Terasaki I. Iwakawa M. Nakano T. Tsukuda A. Kobayashi W. (2010). Novel thermoelectric properties of complex transition-metal oxides. Dalton Trans. 39, 1005–1011. 10.1039/b914661j 20066184

[B30] Troyanchuk I. O. Bushinsky M. V. Tereshko N. V. Lanovsky R. A. Sikolenko V. V. Ritter С. (2019). Ferromagnet-antiferromagnet transition in layered perovskites of Sr_3_YCo_4_O_10.5_ type. Mat. Res. Express 6, 026105. 10.1088/2053-1591/aaef21

[B31] Troyanchuk I. O. Karpinsky D. V. Dobryanskiĭ V. M. Chobot A. N. Chobot G. M. Sazonov A. P. (2009). Magnetic transformations in the Sr_0.78_Y_0.22_Co_1−x_Fe_x_O_3−γ_ system with a perovskite structure. J. Exp. Theor. Phys. 108, 428–434. 10.1134/S1063776109030078

[B32] Tsuruta A. Kawasaki S. Mikami M. Kinemuchi Y. Masuda Y. Fujita A. (2020). Co-substitution effect in room-temperature ferromagnetic oxide Sr_3.1_Y_0_.9Co4O10.5. Mater. (Basel) 13, 2301. 10.3390/ma13102301 PMC7288105 32429414

[B33] Vinila V. S. Isac J. (2022). Synthesis and structural studies of superconducting perovskite GdBa_2_Ca_3_Cu_4_O_10.5+δ_ nanosystems. Des. Fabr. Charact. Multifunct. Nanomater., 319–341. 10.1016/b978-0-12-820558-7.00022-4

[B34] Wu Y. Li L. Chu B. Yi Y. Qin Z. Fan M. (2018). Catalytic reduction of NO by CO over B-site partially substituted LaM_0.25_Co_0.75_O_3_ (M = Cu, Mn, Fe) perovskite oxide catalysts: The correlation between physicochemical properties and catalytic performance. Appl. Catal. A General 568, 43–53. 10.1016/j.apcata.2018.09.022

[B35] Yoshida S. Kobayashi W. Nakano T. Terasaki I. Matsubayashi K. Uwatoko Y. (2009). Chemical and physical pressure effects on the magnetic and transport properties of the A-site ordered perovskite Sr_3_YCo_4_O_10.5_ . J. Phys. Soc. Jpn. 78, 094711–094715. 10.1143/JPSJ.78.094711

[B36] Zhao J. Chen D. J. Shao Z. P. Liu S. M. (2010). Effect of CuO additive on the sintering and performance of niobium-doped strontium cobaltite as oxygen separation membranes. Sep. Purif. Technol. 74, 28–37. 10.1016/j.seppur.2010.05.004

